# An Analytical Thermal Buckling Model for Semiconductor Chips on a Substrate

**DOI:** 10.3390/mi14112025

**Published:** 2023-10-30

**Authors:** Guangping Gong, Dian Xu, Sijun Xiong, Fangyu Yi, Chengbo Wang, Rui Li

**Affiliations:** 1 State Key Laboratory of Structural Analysis, Optimization and CAE Software for Industrial Equipment, Department of Engineering Mechanics, International Research Center for Computational Mechanics, Dalian University of Technology, Dalian 116024, China; 2 Department of Strength, AVIC Shenyang Aircraft Design and Research Institute, Shenyang 110035, China

**Keywords:** thermal buckling, semiconductor chip, symplectic superposition, analytical solution

## Abstract

Semiconductor chips on a substrate have a wide range of applications in electronic devices. However, environmental temperature changes may cause mechanical buckling of the chips, resulting in an urgent demand to develop analytical models to study this issue with high efficiency and accuracy such that safety designs can be sought. In this paper, the thermal buckling of chips on a substrate is considered as that of plates on a Winkler elastic foundation and is studied by the symplectic superposition method (SSM) within the symplectic space-based Hamiltonian system. The solution procedure starts by converting the original problem into two subproblems, which are solved by using the separation of variables and the symplectic eigenvector expansion. Through the equivalence between the original problem and the superposition of subproblems, the final analytical thermal buckling solutions are obtained. The SSM does not require any assumptions of solution forms, which is a distinctive advantage compared with traditional analytical methods. Comprehensive numerical results by the SSM for both buckling temperatures and mode shapes are presented and are well validated through comparison with those using the finite element method. With the solutions obtained, the effects of the moduli of elastic foundations and geometric parameters on critical buckling temperatures and buckling mode shapes are investigated.

## 1. Introduction

Semiconductor chips have garnered significant scholarly attention due to their indispensable engineering applications in stretchable electronics [1], flexible devices [2], microelectromechanical systems [3], etc. It is imperative to carefully consider the mechanical properties of such devices for better structural designs since semiconductor chips may undergo buckling under a temperature rise in service [4,5], which may cause structural instabilities and failures. Recent advancements have emerged in the field of semiconductor chips to address the issue of thermally deduced buckling, among which thermoelectric coolers [6], microprocessors [7], light-emitting diodes [8], and microchannel heat sinks [9] have gained popularity.

**Figure 1:**
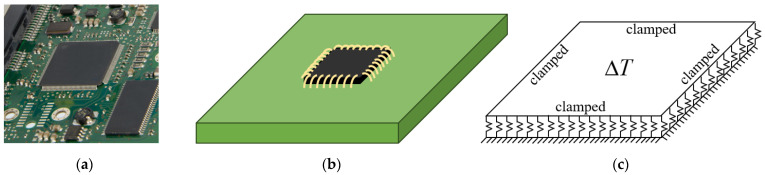
(**a**) Semiconductor chips on a circuit board. (**b**) Schematic diagram of the physical model corresponding to (**a**). (**c**) Equivalent thermal buckling problem of a plate on a Winkler foundation.

Figure 1a illustrates a commonly encountered configuration in semiconductor chip applications, where the chip is bonded to a circuit board. The corresponding physical model is illustrated in Figure 1b and constitutes a challenging three-dimensional problem to solve. To analyze the mechanical behavior of such a structure, an effective way is to consider the chip as a thin plate and the substrate as an elastic foundation, as illustrated in Figure 1c. The heat released by the chip can be equivalent to a uniform temperature field. To better describe the chip bonded to the substrate, the boundary conditions (BCs) are equivalent to fully clamped BCs, which are typically non-Lévy-type. Among various elastic foundation models, the Winkler foundation model stands out [10,11]. In this case, the problem can be modeled as the thermal buckling of a thin plate on a Winkler foundation, which is reduced to solving the higher-order partial differential equations with prescribed BCs. Details appear in Section 2.

A variety of numerical and approximate methods can be employed to address the above issue. Raju and Rao [12] investigated the thermal post-buckling behavior of thick functionally graded microplates attached to elastic foundations using the finite element method (FEM). They evaluated the influence of the foundation stiffness on this problem and concluded that increased foundation stiffness reduces the nonlinearity effect. Shen [13] analyzed the thermal post-buckling behavior of simply supported orthotropic plates attached to elastic foundations by a two-step perturbation technique, and the simulation results clearly demonstrated the significant influence of small-scale parameters on compressive buckling. Noroozi and Jiang [14] utilized the finite difference method to investigate the buckling of functionally graded material semiconductor chips resting on Winkler foundations. The results indicate that the accumulation of wrinkles near the system’s weaker areas displays a notable level of sensitivity due to their lower stiffness. Srinivasan et al. [15] employed an incremental spectral method for solving the buckling of plates on nonlinear foundations under cyclic loading, with the effects of different foundation models discussed in detail. Zhang et al. [16] studied the post-buckling behavior of graphene sheets films on Pasternak-type foundations in a thermal environment using the element-free kp-Ritz method. The results highlight the significant influence of the BCs, graphene sheet film geometry, and elastic foundation on the thermal buckling behavior. Mansouri and Shariyat [17] conducted a buckling analysis of general quadrilateral orthotropic auxetic functionally graded material plates on Winkler–Pasternak elastic foundations in a thermal environment via the differential quadrature method, followed by a comprehensive discussion of the effects of plate parameters and foundation stiffness. Shahrestani et al. [18] applied the isoparametric spline finite strip method to conduct elastic buckling analysis of thin functionally graded material plates, with simulation results indicating that the presence of an elastic foundation leads to an increase in the critical buckling load of the plates. Zenkour and Sobhy [19] presented the trigonometric solutions for the thermal buckling analysis of simply supported functionally graded material plates subjected to different types of temperature loads on Winkler–Pasternak foundations. Zenkour and Radwan [20] presented a quasi-3D model for hygrothermal and mechanical buckling analysis of functionally graded plates resting on two-parameter Pasternak foundations, investigating the influence of foundation parameters and concluding that the presence of the foundations results in an increase in the critical buckling temperature. Zhang and Zhou [21] used the multi-term Ritz method for the mechanical and thermal post-buckling of functionally graded material plates with two opposite supported edges on nonlinear elastic foundations based on higher-order shear deformation theory, followed by a noteworthy conclusion that the nonlinear elastic foundation has a limited impact during the pre-buckling and initial post-buckling state, but its significance increases as deflection escalates in the deep post-buckling state. Shen [22] developed a deflection-type perturbation technique for thermal post-buckling analysis of a shear-deformable plate on a two-parameter elastic foundation based on the Reissner–Mindlin plate theory, with the accuracy of results verified through the comparison with the previous literature. Kiani et al. [23] presented three distinct approximate methods for the thermal buckling analysis of fully clamped thin plates attached to two-parameter Pasternak elastic foundations to obtain closed-form solutions and discussed the effects of geometric parameters and foundation parameters in detail. Cong et al. [24] analyzed the nonlinear thermomechanical buckling and post-buckling problems of porous functionally graded plates with simply supported boundaries using the Galerkin method based on higher-order shear deformation theory. The simulation results revealed that the impact of geometrical parameters and elastic foundations on buckling loads is clearly evident. Duc and Cong [25] utilized the Galerkin method for the analysis of nonlinear thermal post-buckling of imperfect eccentrically stiffened functionally graded plates with simply supported boundaries on a Pasternak elastic foundation and discussed how the buckling and post-buckling loading capacities are affected by the geometric properties, temperature, and elastic foundation. Dung and Nga [26] used the Galerkin method to analyze the nonlinear buckling and post-buckling behavior of eccentrically stiffened functionally graded material plates with simply supported boundaries resting on Pasternak elastic foundations, with the influence of the thermal element, geometric parameters, and foundation stiffness for the thermal buckling and post-buckling problems being evaluated. It was concluded that the effect of the nonlinearity is decreased with the increase in foundation stiffness. Gunda [27] analyzed the thermal post-buckling of a homogeneous, isotropic, square plate attached to a Winkler-type elastic foundation by using the Rayleigh–Ritz method, and verified the accuracy and robustness of the results through a comparison with nonlinear finite element formulation outcomes acquired via an equilibrium path approach.

Although numerical methods can effectively solve a variety of engineering problems within an acceptable margin of error, analytical methods are still vital since they not only provide benchmark solutions but also are helpful for rapid parameter analysis. Bouazza et al. [28] conducted a thermal buckling analysis of nanoplates on a Winkler–Pasternak elastic foundation via the Navier method. Their closed-form solution demonstrated excellent agreement with previously reported solutions in the literature, and it revealed that an increase in the nonlocal parameter coefficient and nonlinear temperature rise leads to a growth of the critical thermal buckling load. Dong et al. [29] investigated the buckling of an infinitely long laminated composite plate on a tensionless elastic foundation based on a one-dimensional analytical method and presented the validity and accuracy of the numerical results compared with the FEM. Naderi and Saidi [30] presented an analytical solution for the buckling of a simply supported functionally graded annular sector plate on an elastic foundation using the energy method. Comprehensive results with different BCs and foundation parameters were presented, and the effects were discussed using the simulation results. Zhang et al. [31] applied the finite integral transform approach for the analytical thermal buckling of plates with simply supported and clamped boundaries and found that the analytical solutions agree well with the solutions obtained by the finite element method. This suggests that the approach has a wide prospect for providing better design for orthotropic plates and other complex structures. Akavci [32] presented Navier-type solutions for the thermal buckling analysis of rigidly fixed simply supported functionally graded plates based on a higher-order hyperbolic shear deformation theory and concluded that with an increase in the aspect ratio or an increase in the thickness-to-length ratio of the plates, the critical buckling temperatures increase. The effects of foundation parameters were also investigated. Yaghoobi and Torabi [33] presented Lévy-type solutions for the buckling analysis of functionally graded material plates on two-parameter Pasternak foundations under uniform, linear, and nonlinear temperature distributions using the power of the series Frobenius method and gave various numerical solutions of different types of thermal loads, geometric parameters, BCs, and foundation parameters. Kiani and Eslami [34] presented an exact analytical solution to study the thermal buckling behavior of heated functionally graded material plates with various boundaries on Winkler elastic foundations, with the effects of both the elastic foundation coefficient and the thermal loading type on the critical buckling temperature of plates being explored.

It is noted that most existing analytical methods are limited to yield only Lévy-type (including Navier-type) solutions for plates with simply supported BCs, leading to an urgent demand for the exploration of new analytical solutions for non-Lévy-type plates. The symplectic superposition method (SSM), incorporating the symplectic approach pioneered by Yao et al. [35] and the superposition technique, has been developed by Li et al. [36,37,38,39,40,41]. The SSM is implemented within the Hamiltonian system instead of the Lagrangian system, which enables the application of mathematical techniques like the separation of variables and symplectic eigenvector expansion within the symplectic framework. With the primary idea of converting the original problem into several subproblems, the solution procedure is carried out without any assumptions of solution forms. The SSM has been applied to solve bending [37,38], free vibration [36,40], and buckling [39,41] problems of non-Lévy-type plates and shells; however, no previous studies have reported on the thermal buckling solutions of plates on elastic foundations using the same method due to the complex mathematical scenario.

In this paper, with the SSM, the thermal buckling of a semiconductor chip on a substrate, which is treated as a thin plate on a Winkler foundation, is studied. The rest of the paper is organized as follows: The governing equation for the thermal buckling of the thin plate in the Hamiltonian system is introduced in Section 2. The analytical thermal buckling solutions of the plate are deduced in Section 3. In Section 4, the convergence study as well as comprehensive numerical and graphic results are presented. The analytical solutions obtained by the SSM are compared with the FEM numerical solutions via ABAQUS software Version 6.13 to verify the accuracy of the former. The effects of the foundation modulus and geometric parameters on the critical buckling temperature and buckling mode shape are also investigated. Conclusions are shown in Section 5. The work presented in this paper contributes to enhancing our understanding of the impact of geometric and environmental conditions on critical buckling temperatures and the buckling modes of semiconductor chips on substrates. Moreover, the analytical solutions obtained can be effectively utilized to facilitate the structural optimization designs for next-generation chip devices.

## 2. Governing Equations for Thermal Buckling of a Thin Plate on a Winkler Foundation within the Hamiltonian Framework

**Figure 2:**
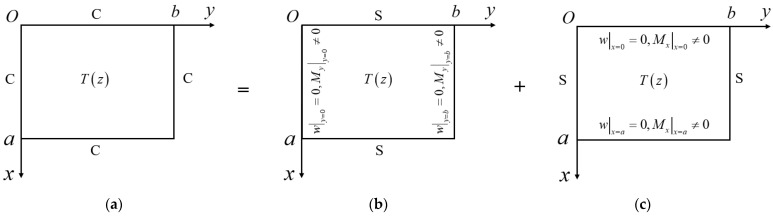
Schematic diagram of symplectic superposition for a fully clamped rectangular plate under thermal buckling. (**a**) Original problem. (**b**) First subproblem. (**c**) Second subproblem.

As shown in Figure 1c, a fully clamped plate on a Winkler foundation in the thermal environment is considered. The schematic diagram of the thermal buckling problem is shown in Figure 2a, where the fully clamped rectangular plate has length a along the Ox axis, width b along the Oy axis, and thickness h perpendicular to the xOy plane. The origin of the coordinate system is located at a corner of the plate. Applying the abbreviation “S” for simply supported and “C” for clamped, the plates under specific BCs will be denoted using a counterclockwise four-letter nomenclature, beginning from the edge at y=b, referring to the coordinate system in Figure 2a.

The governing equations for the thermal buckling of the thin plate are

(1) $$\begin{array}{l} \frac{\partial M_{x}}{\partial x}+\frac{\partial M_{xy}}{\partial y}-Q_{x}=0\\ \frac{\partial M_{y}}{\partial y}+\frac{\partial M_{xy}}{\partial x}-Q_{y}=0\\ \frac{\partial Q_{x}}{\partial x}+\frac{\partial Q_{y}}{\partial y}+N_{x}\frac{\partial^{2}w}{\partial x^{2}}+N_{y}\frac{\partial^{2}w}{\partial y^{2}}+2N_{xy}\frac{\partial^{2}w}{\partial x\partial y}-kw=0 \end{array}$$

where the internal forces are

(2) $$\begin{array}{l} M_{x}=-D\left(\frac{\partial^{2}w}{\partial x^{2}}+v\frac{\partial^{2}w}{\partial y^{2}}\right)+M_{T}\\ M_{y}=-D\left(\frac{\partial^{2}w}{\partial y^{2}}+v\frac{\partial^{2}w}{\partial x^{2}}\right)+M_{T}\\ M_{xy}=-D\left(1-v\right)\frac{\partial^{2}w}{\partial x\partial y}\\ Q_{x}=-D\frac{\partial }{\partial x}\nabla^{2}w\\ Q_{y}=-D\frac{\partial }{\partial y}\nabla^{2}w \end{array}$$

and the equivalent shearing forces are

(3) $$\begin{array}{l} V_{x}=Q_{x}+\frac{\partial M_{xy}}{\partial y}+N_{x}\frac{\partial w}{\partial x}\\ V_{y}=Q_{y}+\frac{\partial M_{xy}}{\partial x}+N_{y}\frac{\partial w}{\partial y} \end{array}$$

where $M_{x}$ and $M_{y}$ are the bending moments about the Oy axis and Ox axis, respectively; $M_{xy}$ is the twisting moment; $Q_{x}$ and $Q_{y}$ are the transverse shear forces perpendicular to the Ox axis and Oy axis, respectively; $N_{x}=N_{y}=-E\alpha \Delta Th/\left(1-\nu \right)$ are the thermally induced membrane forces; $M_{T}=-\int_{-h/2}^{h/2}E\alpha \Delta T/\left(1-\nu \right)zdz$ is the thermally induced resultant moments; w is the modal displacement; h is the thickness of the plate; k is the Winkler foundation modulus; $D=Eh^{3}/\left[12\left(1-\nu^{2}\right)\right]$ is the flexural stiffness; E is the Young’s modulus; and ν is the Poisson’s ratio. It is noted that $N_{xy}$ comes to be zero for special plane-stress-reduced stiffness coefficients, and $M_{T}$ comes to be zero due to the uniform temperature distribution.

By introducing the thermal buckling problem of the plate into the Hamiltonian system, the governing equation is obtained as [41]

(4) $$\frac{\partial Z}{\partial y}=HZ$$

where $H=\left[\begin{array}{cc} F & G\\ Q & -F^{T} \end{array}\right]$, $F=\left[\begin{array}{cc} 0 & 1\\ -\nu \partial^{2}/\partial x^{2} & 0 \end{array}\right]$, $G=\left[\begin{array}{cc} 0 & 0\\ 0 & -1/D \end{array}\right]$, $Q=\left[\begin{array}{cc} -D\left(1-\nu^{2}\right)\partial^{4}/\partial x^{4}+N_{x}\partial^{2}/\partial x^{2}-k & 0\\ 0 & 2D\left(1-\nu \right)\partial^{2}/\partial x^{2}-N_{y} \end{array}\right]$, and $Z={\left[w,\theta_{y},T_{y},M_{y}\right]}^{T}$. It is found that $T_{y}=-V_{y}$. The Hamiltonian operator matrix H satisfies $H^{T}=JHJ$, where $J=\left(\begin{array}{cc} 0 & I_{2}\\ -I_{2} & 0 \end{array}\right)$ is a symplectic matrix in which $I_{2}$ is a 2×2 identity matrix. Therefore, Equation (4) is confirmed as the Hamiltonian system-based governing equation.

## 3. Analytical Thermal Buckling Solutions of CCCC Plates by the SSM

In this section, the thermal buckling problem of a fully clamped rectangular thin plate is explained in detail. Firstly, the original problem is divided into two fundamental subproblems, each of which is based on a plate with simply supported BCs. Secondly, each subproblem is deduced without any assumptions of the solution forms by the techniques of separating variables and symplectic eigenvector expansion. Finally, the analytical solution of the original problem is obtained through the superposition of subproblems’ solutions.

The original problem (Figure 2a) is divided into two subproblems by the SSM, as shown in Figure 2b,c. In Figure 2b, the plate is simply supported at x=0, x=a, y=0, and y=b with the non-zero bending moment ${M_{y}|}_{y=0}$ and ${M_{y}|}_{y=b}$ at y=0 and y=b, respectively. In Figure 2c, the plate of the same size is simply supported at x=0, x=a, y=0, and y=b with the non-zero bending moment ${M_{x}|}_{x=0}$ at x=0 and the non-zero bending moment ${M_{x}|}_{x=a}$ at x=a.

For the first subproblem, the separation of variables is implemented in the symplectic space such that

(5) Z(x,y)=X(x)Y(y)

where the vector $X\left(x\right)={\left[w\left(x\right),\theta_{y}\left(x\right),T_{y}\left(x\right),M_{y}\left(x\right)\right]}^{T}$ and the function Y(y) depend only on x and y, respectively.

Substituting Equation (5) into Equation (4) we obtain the eigenvalue problem

(6) HX(x)=μX(x)

and an ordinary differential equation

(7) $$\frac{dY\left(y\right)}{dy}=\mu Y\left(y\right)$$

where μ is the eigenvalue.

The eigenvalue problem is solved according to the BCs in the x-direction:

(8) $${w|}_{x=0}=0,{M_{x}|}_{x=0}=0,{w|}_{x=a}=0,{M_{x}|}_{x=a}=0$$

The eigenvalues are

(9) $$\begin{array}{l} \mu_{n1}=-\mu_{n2}=\sqrt{\frac{N_{y}}{2D}-\alpha_{n}^{2}-\frac{\sqrt{-4Dk+N_{y}^{2}+4DN_{x}\alpha_{n}^{2}-4DN_{y}\alpha_{n}^{2}}}{2D}}\\ \mu_{n3}=-\mu_{n4}=\sqrt{\frac{N_{y}}{2D}-\alpha_{n}^{2}+\frac{\sqrt{-4Dk+N_{y}^{2}+4DN_{x}\alpha_{n}^{2}-4DN_{y}\alpha_{n}^{2}}}{2D}} \end{array}$$

where $\alpha_{n}=n\pi /a\left(n=1,2,3,\cdots \right)$. The eigenvectors corresponding to the eigenvalues are

(10) $$X_{ni}\left(x\right)=\sin\left(\alpha_{n}x\right){\left[1,\mu_{ni},-\mu_{ni}\left(N_{y}+D\left(2-v\right)\alpha_{n}{}^{2}-D\mu_{ni}^{2}\right),D\left(\nu \alpha_{n}{}^{2}-\mu_{ni}^{2}\right)\right]}^{T}$$

According to the symplectic eigenvector expansion [35], the state vector Z can be expanded as

(11) $$Z=\sum_{n=1}^{\infty }\sum_{i=1}^{4}C_{ni}X_{ni}\left(x\right)e^{\mu_{ni}y}$$

where $C_{ni}\left(i=1,2,3,4;n=1,2,3,\cdots \right)$ are the constants determined by the BCs in the y-direction:

(12) $${w|}_{y=0}=0,{M_{y}|}_{y=0}=\sum_{n=1}^{\infty }E_{n}\sin\left(\alpha_{n}x\right),{w|}_{y=b}=0,{M_{y}|}_{y=b}=\sum_{n=1}^{\infty }F_{n}\sin\left(\alpha_{n}x\right)$$

The final solution of the governing equations expressed in terms of the undetermined coefficients $E_{n}$ and $F_{n}$ for the first subproblem is

(13) $$\begin{array}{ll} w_{1}\left(x,y\right)= & \overset{\infty }{\underset{n=1,2,3,\cdots }{\sum }}\frac{\sin\left(\alpha_{n}x\right)}{D\left(\mu_{n1}^{2}-\mu_{n3}^{2}\right)}\\ & \times \{\left\{\mathrm{csch}\left(b\mu_{n1}\right)\mathrm{sh}\left[\left(b-y\right)\mu_{n3}\right]-\mathrm{csch}\left(b\mu_{n1}\right)\mathrm{sh}\left[\left(b-y\right)\mu_{n1}\right]\right\}E_{n}\\ & +\left[\mathrm{csch}\left(b\mu_{n3}\right)\mathrm{sh}\left(y\mu_{n3}\right)-\mathrm{csch}\left(b\mu_{n1}\right)\mathrm{sh}\left(y\mu_{n1}\right)\right]F_{n}\} \end{array}$$

For the second subproblem, the coordinate transformation is used to replace x(y), a(b), and $N_{x}\left(N_{y}\right)$ with y(x), b(a), and $N_{y}\left(N_{x}\right)$, respectively. The non-zero bending moments ${M_{x}|}_{x=0}$ and ${M_{x}|}_{x=a}$ are expanded as $\sum_{n=1}^{\infty }G_{n}\sin\left(n\pi y/b\right)$ and $\sum_{n=1}^{\infty }H_{n}\sin\left(n\pi y/b\right)$, respectively. The solution of the second subproblem is

(14) $$\begin{array}{ll} w_{2}\left(x,y\right)= & \overset{\infty }{\underset{n=1,2,3,\cdots }{\sum }}\frac{\sin\left(\beta_{n}y\right)}{D\left({\hat{\mu }}_{n1}^{2}-{\hat{\mu }}_{n3}^{2}\right)}\\ & \times \{\left\{-\mathrm{csch}\left(a{\hat{\mu }}_{n1}\right)\mathrm{sh}\left[\left(a-x\right){\hat{\mu }}_{n1}\right]+\mathrm{csch}\left(a{\hat{\mu }}_{n3}\right)\mathrm{sh}\left[\left(a-x\right){\hat{\mu }}_{n3}\right]\right\}G_{n}\\ & +\left[-\mathrm{csch}\left(a{\hat{\mu }}_{n1}\right)\mathrm{sh}\left(x{\hat{\mu }}_{n1}\right)+\mathrm{csch}\left(a{\hat{\mu }}_{n3}\right)\mathrm{sh}\left(x{\hat{\mu }}_{n3}\right)\right]H_{n}\} \end{array}$$

where $\beta_{n}=n\pi /b$, ${\hat{\mu }}_{n1}=\sqrt{N_{x}/2D-\beta_{n}^{2}-\sqrt{-4Dk+N_{x}^{2}+4DN_{y}\beta_{n}^{2}-4DN_{x}\beta_{n}^{2}}/2D}$, and ${\hat{\mu }}_{n3}=\sqrt{N_{x}/2D-\beta_{n}^{2}+\sqrt{-4Dk+N_{x}^{2}+4DN_{y}\beta_{n}^{2}-4DN_{x}\beta_{n}^{2}}/2D}$.

To make the superposition of the two subproblems equivalent to the original problem, the superposition of the subproblems’ solutions must fulfill the actual BCs. For the original problem, the BCs are

(15) $$\begin{array}{l} {\sum_{i=1}^{2}\frac{\partial w_{i}}{\partial x}|}_{x=0,a}=0\\ {\sum_{i=1}^{2}\frac{\partial w_{i}}{\partial y}|}_{y=0,b}=0 \end{array}$$

For y=0, we have

(16) $$\begin{array}{l} \frac{\mu_{n1}\coth\left(b\mu_{n1}\right)-\mu_{n3}\coth\left(b\mu_{n3}\right)}{D\left(\mu_{n1}^{2}-\mu_{n3}^{2}\right)}E_{n}+\frac{-\mu_{n1}\mathrm{csch}\left(b\mu_{n1}\right)+\mu_{n3}\mathrm{csch}\left(b\mu_{n3}\right)}{D\left(\mu_{n1}^{2}-\mu_{n3}^{2}\right)}F_{n}\\ +\sum_{m=1}^{\infty }\frac{2a^{2}mn\pi^{2}}{bD\left(n^{2}\pi^{2}+a^{2}{\hat{\mu }}_{n1}^{2}\right)\left(n^{2}\pi^{2}+a^{2}{\hat{\mu }}_{n3}^{2}\right)}\left[G_{m}-H_{m}\cos\left(n\pi \right)\right]=0 \end{array}$$

for n=1,2,3,⋯. For y=b, we have

(17) $$\begin{array}{l} \frac{\mu_{n1}\mathrm{csch}\left(b\mu_{n1}\right)-\mu_{n3}\mathrm{csch}\left(b\mu_{n3}\right)}{D\left(\mu_{n1}^{2}-\mu_{n3}^{2}\right)}E_{n}+\frac{-\mu_{n1}\coth\left(b\mu_{n1}\right)+\mu_{n3}\coth\left(b\mu_{n3}\right)}{D\left(\mu_{n1}^{2}-\mu_{n3}^{2}\right)}F_{n}\\ +\sum_{m=1}^{\infty }\cos\left(m\pi \right)\frac{2a^{2}mn\pi^{2}}{bD\left(n^{2}\pi^{2}+a^{2}{\hat{\mu }}_{n1}^{2}\right)\left(n^{2}\pi^{2}+a^{2}{\hat{\mu }}_{n3}^{2}\right)}\left[G_{m}-H_{m}\cos\left(n\pi \right)\right]=0 \end{array}$$

for n=1,2,3,⋯. For x=0, we have

(18) $$\begin{array}{l} \frac{{\hat{\mu }}_{n1}\coth\left(a{\hat{\mu }}_{n1}\right)-{\hat{\mu }}_{n3}\coth\left(a{\hat{\mu }}_{n3}\right)}{D\left({\hat{\mu }}_{n1}^{2}-{\hat{\mu }}_{n3}^{2}\right)}G_{n}+\frac{-{\hat{\mu }}_{n1}\mathrm{csch}\left(a{\hat{\mu }}_{n1}\right)+{\hat{\mu }}_{n3}\mathrm{csch}\left(a{\hat{\mu }}_{n3}\right)}{D\left({\hat{\mu }}_{n1}^{2}-{\hat{\mu }}_{n3}^{2}\right)}H_{n}\\ +\sum_{m=1}^{\infty }\frac{2b^{2}mn\pi^{2}}{aD\left(n^{2}\pi^{2}+b^{2}\mu_{n1}^{2}\right)\left(n^{2}\pi^{2}+b^{2}\mu_{n3}^{2}\right)}\left[E_{m}-F_{m}\cos\left(n\pi \right)\right]=0 \end{array}$$

for n=1,2,3,⋯. For x=a, we have

(19) $$\begin{array}{l} \frac{{\hat{\mu }}_{n1}\mathrm{csch}\left(a{\hat{\mu }}_{n1}\right)-{\hat{\mu }}_{n3}\mathrm{csch}\left(a{\hat{\mu }}_{n3}\right)}{D{\hat{\mu }}_{n1}^{2}-D{\hat{\mu }}_{n3}^{2}}G_{n}+\frac{-{\hat{\mu }}_{n1}\coth\left(a{\hat{\mu }}_{n1}\right)+{\hat{\mu }}_{n3}\coth\left(a{\hat{\mu }}_{n3}\right)}{D{\hat{\mu }}_{n1}^{2}-D{\hat{\mu }}_{n3}^{2}}H_{n}\\ +\sum_{m=1}^{\infty }\cos\left(m\pi \right)\frac{2b^{2}mn\pi^{2}}{aD\left(n^{2}\pi^{2}+b^{2}\mu_{n1}^{2}\right)\left(n^{2}\pi^{2}+b^{2}\mu_{n3}^{2}\right)}\left[E_{m}-F_{m}\cos\left(n\pi \right)\right]=0 \end{array}$$

for n=1,2,3,⋯.

Therefore, we obtain an infinite system of linear equations regarding the coefficients $E_{n}$, $F_{n}$, $G_{m}$, and $H_{m}$(n=1,2,3,⋯;m=1,2,3,⋯). The fundamental solutions are obtained by the zero determinant of the coefficient matrix. Subsequently, the thermal buckling mode shape solutions can be finally obtained by the superposition of two non-trivial fundamental solutions.

## 4. Comprehensive Benchmark Results

To ensure accurate and reliable benchmarks for subsequent structural designs, the analytical thermal buckling results of semiconductor chips are presented using the SSM and verified with the FEM. All the present numerical and graphical results based on the SSM were achieved through programming in the Mathematica software Version 12.0, and the FEM-based numerical solutions were obtained via the ABAQUS software [42] where the shell element S4R of mesh size b/200 was used for comparison. In the numerical examples, Poisson’s ratio ν=0.33, Young’s modulus E=20 GPa, and the coefficient of thermal expansion $\alpha =1.5\times 10^{-5}/{}^{\circ}C$ are taken, and the thickness-to-width ratio of the plate h/b=0.01 is given.

Firstly, the convergence study for the buckling temperatures of the plates with different aspect ratios, a/b= 1 and 4, on a Winkler foundation with $kb^{4}/D=1000$ was conducted. The benchmark results followed by the FEM are tabulated in Table 1, in which the bold numbers indicate convergent results. Table 1 shows that all numerical results achieve the accuracy of five significant figures when the number of series terms is increased to 20. Therefore, the number of series terms is taken to be 20 to guarantee the accuracy of five significant digits for all results obtained by the SSM.

**Figure 3:**
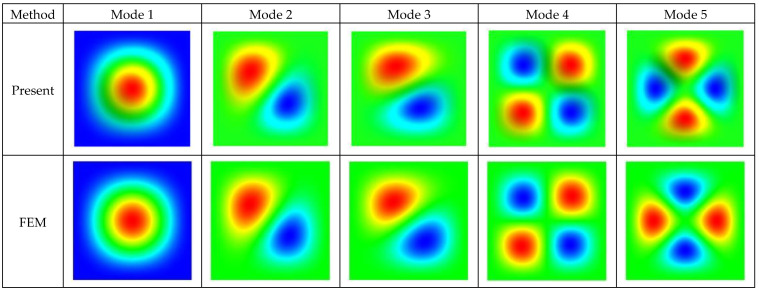
First five thermal buckling mode shapes of a CCCC plate with *a*/*b* = 1 on a Winkler foundation with *kb*^4^/*D* = 1000. The colors of the rainbow spectrum shift from blue to red, indicating a continuous variation in modal displacement values along the axis perpendicular to the *xOy* plane, moving from bottom to top.

Additionally, the first five buckling temperatures of CCCC plates with aspect ratios a/b= 1, 2, 3, and 4 on elastic foundations with different foundation parameters $kb^{4}/D=$ 0, 1000, and 10,000 are given, as shown in Table 2. The maximum relative difference between the results of the current method and the FEM does not exceed 0.50%. Figure 3 plots the first five thermal buckling mode shapes of the CCCC square plate on the Winkler foundation with $kb^{4}/D=1000$. As shown in Table 2 and Figure 3, the present results are in good agreement with those from the FEM, providing strong evidence for the validity of the SSM.

**Figure 4:**
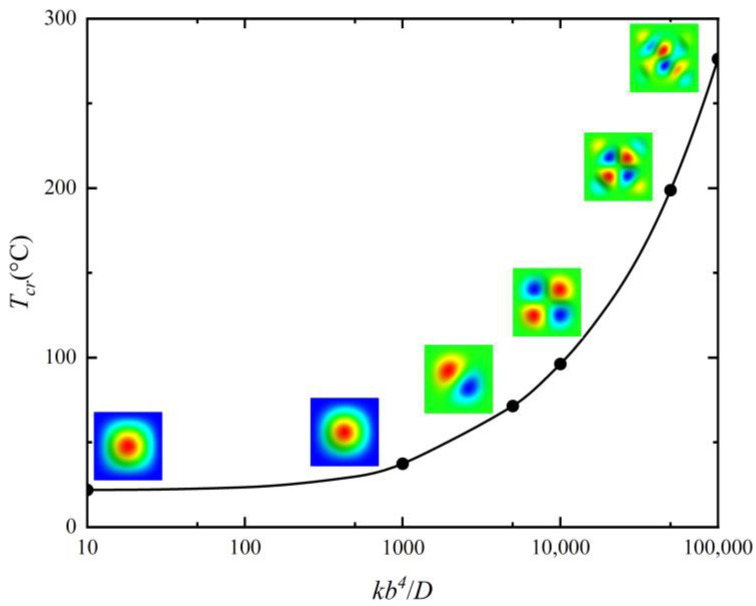
Critical buckling temperatures and corresponding buckling mode shapes of a CCCC plate with *a*/*b* = 1 and *h*/*b* = 0.01 for different foundation parameters *kb*^4^/*D*.

**Figure 5:**
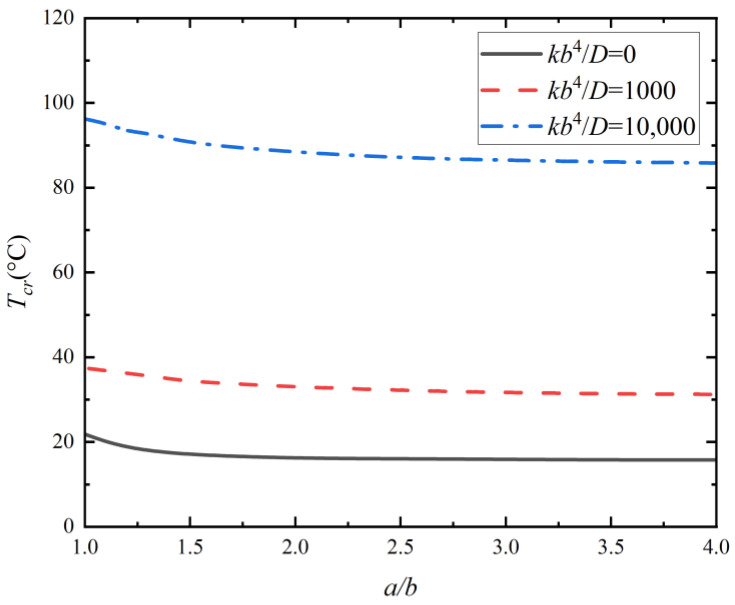
Critical buckling temperature versus aspect ratio, with foundation parameters *kb*^4^/*D* = 0, 1000, and 10,000.

With the analytical solutions, the effects of the moduli of the Winkler foundation and geometric parameters on the critical buckling temperature $T_{cr}$ are investigated, as shown in Figure 4 and Figure 5. The results in Figure 4 illustrate the effects of foundation parameters $kb^{4}/D$ ranging from 1000 to 100,000 on the critical buckling temperature. It is observed that as the Winkler foundation’s modulus increases, the critical buckling temperature and the half wave number of thermal buckling mode shape increase concomitantly, resulting in more complex buckling modes. The results in Figure 5 illustrate the effects of aspect ratio a/b ranging from 1 to 4 on the critical buckling temperature. It is observed that as the aspect ratio increases, the critical buckling temperatures of plates on foundations with different moduli show a continuous decline, gradually transitioning to a smoother downward trend.

## 5. Conclusions

This study gives an analytical model to solve the problem of thermal buckling of semiconductor chips on a substrate within the framework of the Hamiltonian system. The SSM is used to transform the original problem into two subproblems, and the separation of variables and symplectic eigenvector expansion are utilized to solve each subproblem. The buckling temperatures and corresponding mode shapes are determined by the requirement of the equivalence between the original problem and the superposition of the two subproblems. After the fast convergence of the method is verified, comprehensive numerical examples are provided, which can serve as benchmarks. With the analytical solutions, the effects of the moduli of the Winkler foundations and geometric parameters are quantitatively studied. Specifically, it is observed that with the increase in the foundation parameter, both the critical buckling temperature and the half wave number of thermal buckling mode shape increase. Additionally, the critical buckling temperatures of plates continuously decline with an increase in aspect ratio. From the perspective of ensuring safety, a larger foundation modulus is favored and the shape of the semiconductor chips is recommended to be square to protect semiconductor chips from thermal buckling. While recognizing the inherent merits of the current approach in terms of its versatility and accuracy, it is important to acknowledge that, like any other solution method, it has certain limitations. The SSM is primarily tailored for addressing linear partial differential equation problems, posing limitations when encountering nonlinear partial differential equations. To tackle complex issues involving plastic behavior or substantial deformations, it becomes necessary to complement the method with perturbation techniques to effectively linearize the nonlinear equations and subsequently address them as a combination of linear equations. Although this paper focuses on thermal buckling problems of plates on elastic foundations, it is also necessary to mention that the SSM holds the potential for broader applications in the analysis of bending, vibration, and buckling problems associated with similar structures.

## Figures and Tables

**Table 1:** Convergence of the first five buckling temperatures (°C) of CCCC plates on a Winkler foundation with $kb^{4}/D=1000$.

*a*/*b*	Number of Series Terms	Modes				
		1st	2nd	3rd	4th	5th
1	5	37.412	45.358	45.358	57.946	68.008
	10	37.412	45.362	45.362	57.991	68.008
	**15**	**37.412**	**45.362**	**45.362**	**57.991**	**68.008**
	20	37.412	45.362	45.362	57.991	68.008
4	5	27.251	28.276	30.980	31.831	33.547
	10	31.225	31.305	32.943	33.323	35.675
	15	31.227	31.307	32.977	33.329	35.709
	**20**	**31.227**	**31.307**	**32.977**	**33.329**	**35.710**
	25	31.227	31.307	32.977	33.329	35.710

Note: the bold numbers indicate convergent results.

**Table 2:** First five buckling temperatures (°C) of the CCCC plates with h/b=0.01.

*ka*^4^/*D*	*a*/*b*	Method	Modes				
			1st	2nd	3rd	4th	5th
0	1	Present	21.865	38.481	38.481	53.555	64.380
		FEM	21.828	38.369	38.369	53.327	64.080
		Difference	0.17%	0.29%	0.29%	0.43%	0.47%
	2	Present	16.175	17.772	22.840	30.733	34.373
		FEM	16.156	17.747	22.798	30.662	34.290
		Difference	0.12%	0.14%	0.18%	0.23%	0.24%
	3	Present	15.916	16.042	17.057	19.484	23.340
		FEM	15.896	16.024	17.034	19.453	23.296
		Difference	0.12%	0.11%	0.14%	0.16%	0.19%
	4	Present	15.774	15.785	16.297	16.682	18.207
		FEM	15.755	15.765	16.279	16.659	18.179
		Difference	0.12%	0.13%	0.11%	0.14%	0.15%
1000	1	Present	37.412	45.362	45.362	57.991	68.008
		FEM	37.371	45.252	45.252	57.766	67.710
		Difference	0.11%	0.24%	0.24%	0.39%	0.44%
	2	Present	32.992	33.357	39.222	40.327	42.446
		FEM	32.953	33.324	39.156	40.283	42.364
		Difference	0.12%	0.10%	0.17%	0.11%	0.19%
	3	Present	31.646	31.898	34.618	35.471	38.988
		FEM	31.614	31.863	34.583	35.422	38.946
		Difference	0.10%	0.11%	0.10%	0.14%	0.11%
	4	Present	31.227	31.307	32.977	33.329	35.710
		FEM	31.196	31.277	32.939	33.295	35.663
		Difference	0.10%	0.10%	0.12%	0.10%	0.13%
10,000	1	Present	96.233	96.885	98.492	98.492	99.973
		FEM	95.995	96.627	98.251	98.251	99.672
		Difference	0.25%	0.27%	0.25%	0.25%	0.30%
	2	Present	88.350	88.463	91.694	91.937	96.532
		FEM	88.181	88.311	91.500	91.725	96.267
		Difference	0.19%	0.17%	0.21%	0.23%	0.27%
	3	Present	86.486	86.511	90.664	90.751	90.819
		FEM	86.337	86.371	90.467	90.559	90.624
		Difference	0.17%	0.16%	0.22%	0.21%	0.21%
	4	Present	85.824	85.833	88.327	88.426	90.295
		FEM	85.679	85.694	88.157	88.272	90.103
		Difference	0.17%	0.16%	0.19%	0.17%	0.21%

## Data Availability

The data presented in this study are available on reasonable request from the corresponding author.
